# Thermal selectivity of intermolecular versus intramolecular reactions on surfaces

**DOI:** 10.1038/ncomms11002

**Published:** 2016-03-11

**Authors:** Borja Cirera, Nelson Giménez-Agulló, Jonas Björk, Francisco Martínez-Peña, Alberto Martin-Jimenez, Jonathan Rodriguez-Fernandez, Ana M. Pizarro, Roberto Otero, José M. Gallego, Pablo Ballester, José R. Galan-Mascaros, David Ecija

**Affiliations:** 1 IMDEA Nanoscience, c/Faraday 9, Cantoblanco, 28049 Madrid, Spain; 2 Institute of Chemical Research of Catalonia, Barcelona Institute of Science and Technology, Avinguda Països Catalans 16, Tarragona 43007, Spain; 3 Department of Physics, Chemistry and Biology, IFM, Linköping University, 58183 Linköping, Sweden; 4 Departamento de Física de la Materia Condensada, Universidad Autónoma de Madrid, c/Francisco Tomás y Valiente 7, Cantoblanco, 28049 Madrid, Spain; 5 Instituto de Ciencia de Materiales de Madrid, c/ Sor Juana Inés de la Cruz 3, Cantoblanco, 28049 Madrid, Spain; 6 Catalan Institution for Research and Advanced Studies, Passeig Lluis Companys 23, Barcelona 08010, Spain

## Abstract

On-surface synthesis is a promising strategy for engineering heteroatomic covalent nanoarchitectures with prospects in electronics, optoelectronics and photovoltaics. Here we report the thermal tunability of reaction pathways of a molecular precursor in order to select intramolecular versus intermolecular reactions, yielding monomeric or polymeric phthalocyanine derivatives, respectively. Deposition of tetra-aza-porphyrin species bearing ethyl termini on Au(111) held at room temperature results in a close-packed assembly. Upon annealing from room temperature to 275 °C, the molecular precursors undergo a series of covalent reactions via their ethyl termini, giving rise to phthalocyanine tapes. However, deposition of the tetra-aza-porphyrin derivatives on Au(111) held at 300 °C results in the formation and self-assembly of monomeric phthalocyanines. A systematic scanning tunnelling microscopy study of reaction intermediates, combined with density functional calculations, suggests a [2+2] cycloaddition as responsible for the initial linkage between molecular precursors, whereas the monomeric reaction is rationalized as an electrocyclic ring closure.

## Abstract (web summary)

Surface-mediated synthesis of low-dimensional polymers is a promising approach to design materials for targeted applications. Here, the authors introduce surface-confined thermally tunable pathways to select intra- or intermolecular reactions yielding monomeric or lowdimensional polymeric phthalocyanines.

## Graphical abstract

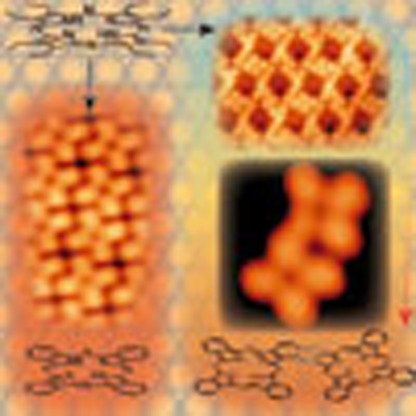

Surface-mediated synthesis of low-dimensional polymers from simple molecular precursors (monomers) under ultra-clean conditions is a rapidly emerging field with relevance for molecular electronics, optoelectronic devices, magnetism, molecular recognition and sensing, catalysis, filtration and membranes^1^,^2^,^3^,^4^,^5^. This promising bottom-up strategy relies on a careful selection of building blocks equipped with functional groups and surfaces, whereby one-dimensional and two-dimensional nanostructures have been engineered and a plethora of new materials is envisioned^6^,^7^,^8^,^9^,^10^,^11^,^12^,^13^,^14^,^15^,^16^,^17^,^18^,^19^,^20^,^21^.

Regarding the molecular precursors, porphyrin and phthalocyanine derivatives are the subject of increasing attention, inspired by scientific curiosity, biological relevance and potential technological impact^22^.

Several chemical reactions have been exploited to produce surface-assisted lateral covalent designs inspected with ultimate spatial resolution^23^. These include cyclocondensation of boronic acids^8^, tetramerization of 1,2,4,5-tetracyanobenzene with iron^16^, Schiff-base reaction of aldehydes and amines to form imine^24^, acylation reactions (yielding polyamides^25^, polyimides^7^ or polyesters^26^), Bergmann cyclization^27^, azide–alkyne cycloadditions^28^,^29^ and carbon–carbon couplings exemplified by alkane polymerizations^30^, the Ullmann^6^,^17^, the Glaser^18^ and the Sonogashira reactions^21^ or dehalogenation reactions followed by radical combination^13^,^14^,^31^. Despite their importance in bulk chemistry and their growing relevance in 3D polymer science, [2+2] cycloaddition reactions have not yet been exploited to produce surface-confined polymeric nanostructures on surfaces.

Importantly, the selectivity of reaction pathways under distinct thermal stimuli, although being a promising strategy to increase synthetic versatility, has remained mostly elusive on surfaces^17^. Here, we introduce surface-confined thermally tunable reaction pathways as a route to select intramolecular versus intermolecular covalent reactions yielding either monomeric phthalocyanines or low-dimensional phthalocyanine polymers, respectively. To this end, we deposit 2,3,7,8,12,13,17,18-octaethyl-5,10,15,20-tetraazaporphyrin (OETAP) under ultra-high vacuum on a pristine Au(111) crystal held at room temperature, forming close-packed supramolecular assemblies. This precursor phase is gently annealed to 300 °C giving rise to quasi-one-dimensional polymers that can be rationalized as phthalocyanine derivatives. Further insights of the polymerization process with high-resolution low-temperature scanning tunnelling microscopy (STM) and state-of-the-art density functional (DFT) calculations reveal that the linkage between two OETAPs is likely to be initiated by a [2+2] cycloaddition, involving the chemical transformations of the peripheral ethyl substituents of two adjacent molecular species. Importantly, by raising the temperature of the substrate prior deposition of the molecular precursors, the intermolecular covalent coupling can be precluded. For substrate temperatures of 300 °C or higher, it is observed that the mechanisms for polymeric growth are blocked and the OETAP species are transformed into individual phthalocyanines, via an electrocyclic ring closure (ERC) reaction, and then self-assembled into islands. DFT simulations support our experimental findings indicating a lower energetic barrier for dimerization as compared with the intramolecular reaction of the monomer. We envision that our results will pave the way for the development of low-dimensional materials exploiting the propensity of molecular precursors equipped with convenient peripheries to undergo tunable covalent reactions under thermal or light stimuli.

## Results

### Self-assembly of OETAP precursors on Au(111)

**Figure 1:**
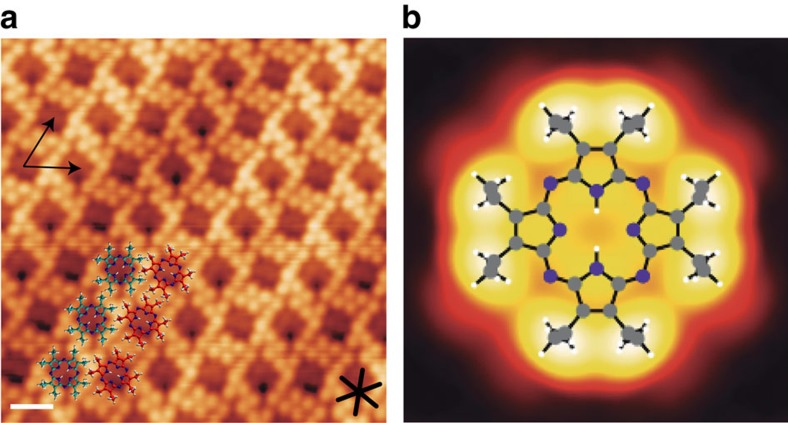
Self-assembly of OETAP on Au(111) after deposition at room temperature. (**a**) High-resolution STM images revealing submolecular features. Black arrows depict the lattice vectors of the rhombic unit cell. Black star shows the close-packed directions of the surface. Superimposed coloured atomistic models address the two distinctly oriented molecular species (green and red, respectively). Tunnelling parameters: *V*_b_=0.5 V, *I*=0.1 nA. Scale bar, 1 nm. (**b**) STM simulated image of an individual OETAP on Au(111) at 0.5 V, with a superimposed ball-and-stick model of the molecular species. Hydrogen, carbon and nitrogen atoms are depicted in white, grey and violet, respectively.

Figure 1a shows a high-resolution STM image of an OETAP array on Au(111) self-assembled after room temperature deposition. Each molecule is visualized as a dim centre, attributed to the macrocyclic core, surrounded by eight brighter protrusions, which are assigned to the ethyl moieties, in excellent agreement with simulated STM images (cf. Fig. 1b and Supplementary Fig. 1) and consistent with surface-confined assemblies of identically substituted porphyrin compounds^32^. The dense-packed architecture displays two distinctly oriented molecular species, related by a ∼16° rotation, which features a rhombic lattice (*a*=14.1±1 Å, *b*=14.1±1 Å, *θ*=60°), and is stabilized by lateral non-covalent interactions between the ethyl substituents.

### Polymerization of OETAP species

**Figure 2:**
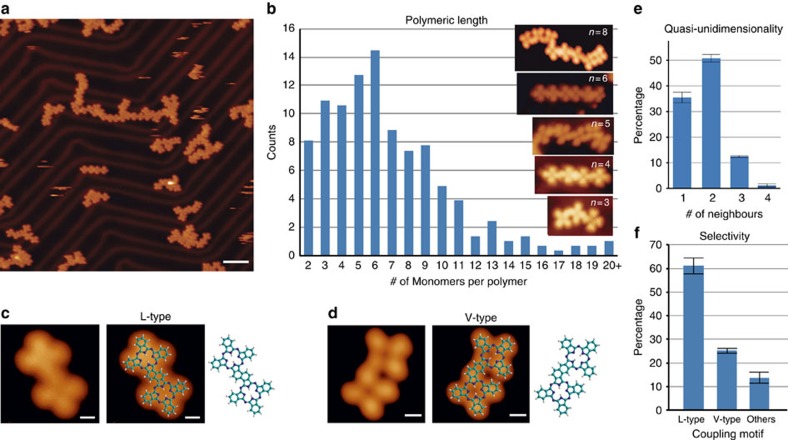
Surface-confined synthesis of phthalocyanine tapes. The deposition of 0.3 ML of OETAP species (precursors) on Au(111) held at room temperature and subsequent gently annealing at 275 °C affords the formation of phthalocyanine polymers. (**a**) Long-range STM image displaying the formation of quasi-unidimensional polymers mainly confined to the fcc regions. (**b**) High-resolution STM images of polymers of distinct size (3, 4, 5, 6 and 8) and histogram of the polymeric length. (**c**,**d**) High-resolution STM image and models of the majority of products between coupled monomers (**c**, L-type and **d**, V-type). (**e**) Histogram of the number of neighbours per monomer, highlighting the quasi-unidimensional feature of the assemblies. (**f**) Histogram of different types of coupling motifs exhibiting selectivity towards L-type-binding motif. Errors bars in **e**,**f** are the standard deviation of the mean. Tunnelling parameters: **a**, *V*_b_=−1.5 V; **b**, *V*_b_=−0.5 V for *n*=3, *V*_b_=−1.5 V for *n*=4, *V*_b_=−1 V for *n*=5, *V*_b_=−1 V for *n*=6, *V*_b_=−1.2 V for *n*=8; **c**, *V*_b_=−1.5 V and **d**, *V*_b_=−0.7 V. Scale bars, **a**, 5 nm; **c**,**d**, 0.5 nm.

Next we explore the polymerization of OETAP precursors on Au(111). After submonolayer deposition at room temperature, gentle annealing of the substrate to 75, 100 and 150 °C results in no appreciable changes. At 225 °C, the polymerization of OETAP species is initiated. High-resolution images of the assemblies after holding the substrate at 275 °C reveal the formation of molecular chains, most of them confined on the fcc regions of Au(111) (cf. Fig. 2a). A careful inspection of these chains allows us to discern submolecular features and, thus, clarify the molecular organization. The tetrapyrrolic macrocyclic cores are now visualized as dim crosses, whereas the ethyl substituents have reacted in two distinct ways: (i) by covalently linking adjacent species through the intermediacy of multiple covalent reactions (cf. below) and (ii) by undergoing an intramolecular ERC reaction. The final result is the formation of polymeric chains, in which the repeating unit (monomer) derives from OETAP. A statistical analysis shows that the average polymeric size is ∼7 monomers, that the most frequent polymer comprises 6 macrocycles and that extended polymers (>20 monomers) can be formed. Importantly, the majority of the monomeric entities are joined together via two covalent structural motifs, denoted L and V (cf. Fig. 2c,d). Both structural motifs are the product of multiple covalent reactions between peripheral ethyl groups from adjacent OETAP precursors, where two molecules face each other. When the two ethyl groups that initiate the intermolecular reaction lie in opposite sides of the OETAP precursors the product is an L-type motif (cf. Fig. 2c and Supplementary Figs 1 and 2 for agreement with DFT simulated image), whereas the V-type motif is the product when the ethyl groups lie in the same side of opposite molecules. In both cases, the resulting polymer can be interpreted as a phthalocyanine tape with locally straight (L) or slightly curved (V) appearance. Notably, the thermally induced polymerization of OETAP precursors into phthalocyanine tapes seems to present a preference for the L-type coupling motif versus the V-type motif (ca 2:1), as seen in Fig. 2f. In addition, polymers are mostly quasi-unidimensional, with over 85% of the monomers connected to only one or two neighbours. The remaining 15% of monomers are connected to three or four neighbours mainly through cross motifs based on L or V coupling schemes.

Regarding the proposed covalent binding motifs, we are certain that the observed molecular chains are polymers and not supramolecular assemblies due to the following reasons: (i) the centre-to-centre distance between macrocycles is too short for a non-covalent interaction and our DFT study of the reaction pathway (see below for details) is fully consistent with the experimental observations; and (ii) using perturbative scanning conditions, the polymeric chains can be displaced as entire units for long distances (over 10 nm) preserving their size and shape, thus revealing a strong covalent interaction between the monomers (cf. Supplementary Fig. 3 and Supplementary Movie 1).

### Transformation of OETAP precursors into phthalocyanines

**Figure 3:**
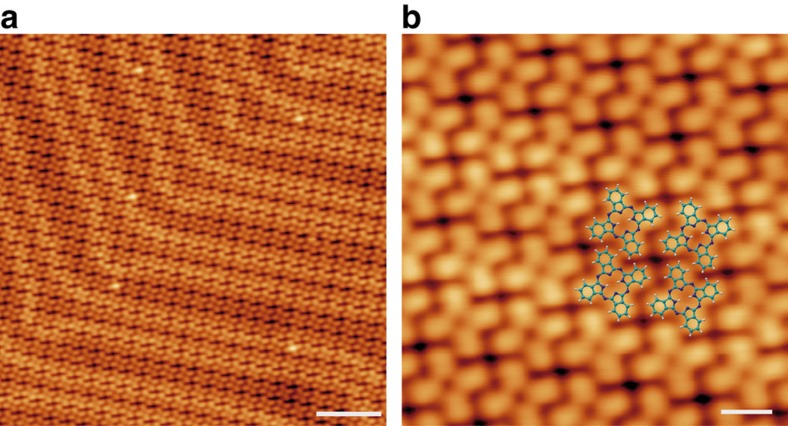
Surface-assisted synthesis and self-assembly of phthalocyanines. The deposition of OETAP species on Au(111) held at 300 °C affords the on-surface chemical transformation of precursors into phthalocyanine species. (**a**) Long-range STM image, revealing the close-packed supramolecular assembly and highlighting the presence of the herringbone reconstruction of Au(111). (**b**) High-resolution STM image and superimposed modelling of the molecular constituents, which matches the reported assembly of 2H-Pc on Au(111). **a**,**b**, *V*_b_=−1 V. Scale bars: **a**, 5 nm; **b**, 1 nm.

To test the feasibility of thermal control of the covalent reaction pathways, OETAP precursors were deposited on pristine Au(111) held at 300 °C. As depicted in Fig. 3, a new scenario is manifested. Instead of polymeric chains, we observe the formation of individual entities, which self-assemble into close-packed arrays based on a square unit cell. High-resolution STM imaging allows us to discern submolecular features of the assemblies, whereby each molecular species presents four bright protrusions and a central void forming a cross-like shape. Both the molecular appearance and the self-assembled architecture are identical to those found after deposition of 2H-Pc (free base phthalocyanine) on Au(111)^33^. Thus, based on our results and a comparison with the literature, we suggest that the deposition of OETAP precursors on Au(111) held at 300 °C gives rise to the formation of 2H-Pc species, thanks to the dehydrogenation and ring closure of their ethyl peripheries, a phenomenon observed for porphyrin derivatives^32^.

### Reaction intermediates during polymerization

To address the reaction mechanisms that could plausibly explain the formation of the covalent phthalocyanine polymers and phthalocyanine monomers, a detailed analysis of the STM images collected during the annealing process was carried out.

**Figure 4:**
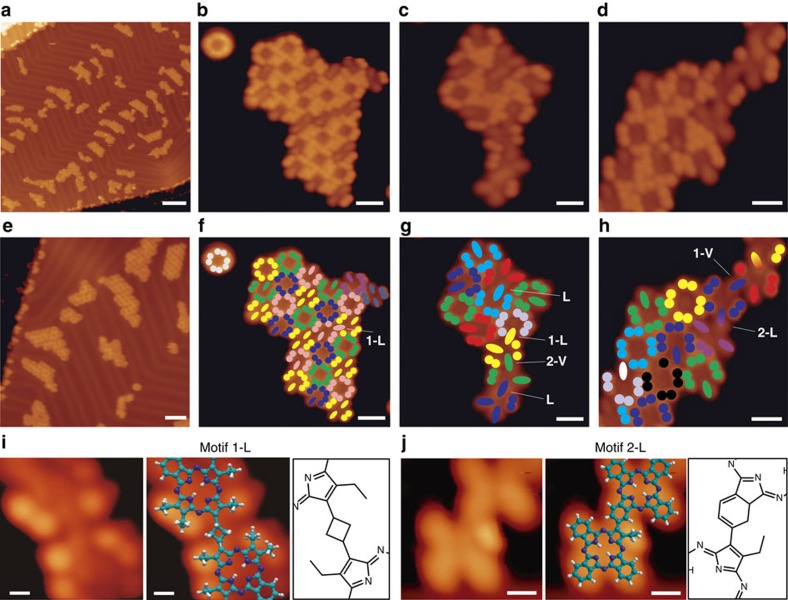
Surface-assisted formation of phthalocyanine derivatives after deposition of OETAP precursors on Au(111). (**a**,**e**) Large-scale STM images showing the formation of patches of initially reacted molecules coexisting with unreacted species. (**b**–**d**) High-resolution STM images and (**f**–**h**) corresponding schematic colouring of the initial steps of reactions giving motifs **1-L**, **1-V**, **2-L**, **2-V** and **L**. (**i**,**j**) Zoom-in of motif **1-L** and **2-L**, and superimposed modelling, together with chemical drawing insets. Coloured filled dots represent ethyl moieties, bi-coloured ellipses show covalent connections between adjacent species, and mono-coloured ellipses represent full electrocyclic ring closures. Tunnelling parameters: **a**, *V*_b_=−0.8 V; **b**, *V*_b_=−0.7 V; **c**, *V*_b_=−0.7 V; **d**, *V*_b_=−0.8 V; **e**, *V*_b_=−1.2 V; **i**, *V*_b_=0.5 V and **j**, *V*_b_=−1.5 V. Scale bars, **a**, 10 nm; **b**, 1.5 nm; **c**, 1.0 nm; **d**, 1.0 nm; **e**, 5 nm; **f**, 1.5 nm; **g**, 1.0 nm; **h**, 1.0 nm; **i**, 0.3 nm and **j**, 0.5 nm.

Figure 4 illustrates reaction intermediates observed after depositing OETAP species holding the substrate at room temperature, subsequently followed by gentle annealing to 225 °C. Long-range STM images show the formation of small islands mainly distributed over the fcc regions of the Au(111) surface (cf. Fig. 4a,e), thus revealing the dissolution of the former OETAPs islands and subsequent self-assembly into smaller patches. High-resolution topographs (cf. Fig. 4b–d) allow us to discern submolecular features and elucidate the molecular packing. For clarity, Fig. 4f–h display a rationalization of the distinct molecular species by superimposing a coloured model. Here, dots and rods of the same colour represent intact ethyl moieties and ERC-reacted ethyl termini of the same molecule, respectively. In addition, bi-coloured rods indicate links between tetrapyrrole molecular units. A minority of unreacted OETAP species, characterized by the eight-dotted appearance described above, are still observed either isolated on the surface or forming part of the supramolecular islands. However, most of the molecular precursors have experienced covalent reactions, giving rise to the reaction intermediates of the polymeric chains. The majority of OETAPs have undergone one or two ring closure reactions (intramolecular electro-cyclizations) that transformed their diethyl-pyrrole units into isoindole components, which are imaged as rods. At this stage of the reaction, some of the OETAPs are also covalently linked to one another, affording distinct covalent bonding motifs, assignable to intermediates of the two main final structures (L and V). **1-L** and **2-L** represent reaction intermediates of the L-type coupling motif (cf. Fig. 4i,j), whereas **1-V** and **2-V** are the corresponding analogous reaction intermediates of the V-type-binding motif. We observe that the intermolecular bond in intermediate **1-L** comprises the reaction between two ethyl moieties in opposite sides of the OETAP precursors (cf. Fig. 4i and Supplementary Fig. 4), keeping the other two ethyl peripheries of the pyrroles intact, which are visualized as bright lobes with the same height as in individual unaltered OETAPs. In **2-L**, the reaction appears to have evolved, involving another ethyl group (cf. Fig. 4j), thus keeping only one of the four initial ethyl groups unreacted. Finally, in **L**, the four ethyl groups have reacted to produce the seed of a polymeric chain.

## Discussion

**Figure 5:**
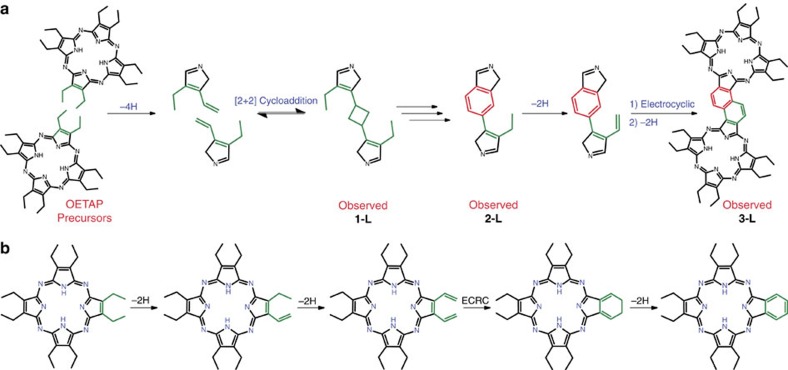
Proposed reaction mechanisms describing the surface-assisted formation of phthalocyanine polymers and monomers on Au(111). (**a**) Reaction steps involving STM-imaged intermediates **1-L** and **2-L** in the phthalocyanine dimer formation (**3-L** reaction product) via L-type linking motif. For a full step-wise reaction mechanism and for the analogous reaction of the V-type motif, see Supplementary Figs 5 and 6. (**b**) Diethyl-pyrrole to isoindole moiety conversion.

Taking into account the initial precursors, the identified intermediates and the final products, we tentatively propose multi-step reaction mechanisms for both pathways, the polymerization of OETAPs into phthalocyanine quasi-unidimensional tapes (cf. Fig. 5a and Supplementary Figs 5 and 6), and the transformation of OETAP species into unsubstituted monomeric phthalocyanines (cf. Fig. 5b).

At the first stage of the polymeric reaction, two OETAPs face each other, positioning four ethyl moieties opposite each other (shown in green in Fig. 5a), which will undergo a series of complex reactions activated by the ramp annealing of the substrate. We propose that the ethyl substituents undergo an initial dehydrogenation reaction to produce an ethenyl residue per molecule, either in s-*cis* or s-*trans* conformation. Two opposite ethenyl groups adequately oriented are susceptible to react via a [2+2] cycloaddition affording intermediate **1-L** (or **-V)**, which is repeatedly observed by STM imaging. Further work is necessary to corroborate this mechanism. The L-to-V ratio in the final coupling motifs (ca. 2:1) corresponds to a preferential ethyl-to-ethenyl dehydrogenation favouring the conformation where the ethenyl group lies s-*trans* to the bond opposite the pyrrolic nitrogen, as shown in Fig. 5a. Intermediate **1-L/V** can be considered as a dimer of two OETAP macrocycles joined together through a four-membered ring, fitting the appropriate angles and bond distances. This type of reaction, thermally forbidden by the Woodward–Hoffmann rules, is unprecedented on metallic surfaces and has only been explored on semiconductor surfaces to covalently functionalize the substrate support, whereby dangling bonds play a major role^34^. Encouragingly, DFT simulations support the tentative [2+2] cycloaddition (vide infra).

To explain intermediate **2-L/V**, also observed by STM imaging, a series of chemical reactions, including the loss of two methylene groups (as ethene), is tentatively suggested (cf. Supplementary Figs 5 and 6, and Supplementary Note 1). An additional dehydrogenation reaction of the remaining ethyl substituent and subsequent 6-π electrocyclization reaction followed by an aromatization process results in the formation of STM-characterized dimer **3**, observed in both L and V conformations, with a naphthalene spacer bridging the two tetrapyrrolic macrocycles.

During the polymerization process, we observe that the ethyl groups not involved in the construction of the covalent bridging spacer undergo a series of dehydrogenation and electrocyclic ring closures, giving rise to isoindole units, and thus providing the final phthalocyanine aspect to the polymers. In short, the dehydrogenation of the β-ethyl groups followed by the intramolecular electrocyclization of the resulting ethenyl resides transformed the 3,4-diethylpyrrole units into isoindole analogues (cf. Fig. 5b). We provide theoretical results (see below for details) supporting our suggestion that an analogous mechanism takes place in the transformation of OETAPs into 2H-Pcs, during the deposition process of the tetraazaporphyrins on Au(111) held at 300 °C, as previously observed for β-ethyl-substituted porphyrins^32^.

To gain additional insight into reaction mechanisms, we performed DFT calculations for a model system on the Au(111) surface. We studied and compared the monomer electro-cyclization and the initial dimerization process. The complete dimerization process involves a complex multi-step sequence of events and a more in-depth analysis of all reaction steps will be accounted for elsewhere. Both the monomer electro-cyclization and the dimerization reaction of monomers require four preliminary dehydrogenation reactions that converted two ethyl substituents into ethenyl residues, with a largest energy barrier of 1.60 eV (cf. Supplementary Fig. 7 for details).

**Figure 6:**
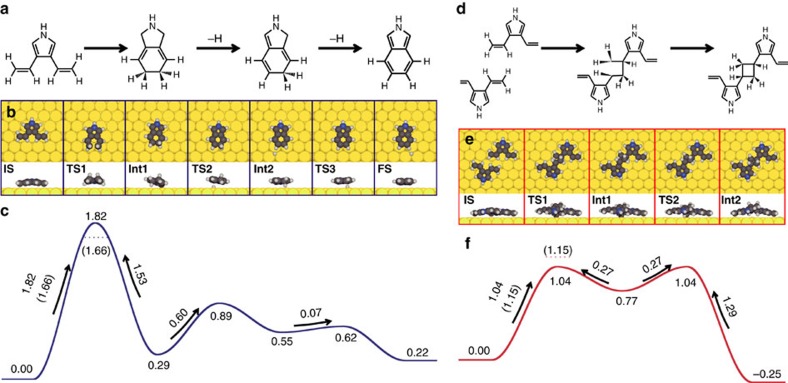
Reaction pathway considerations for a model pyrrole molecule on Au(111). (**a**–**c**) Monomer cyclization. (**d**–**f**) Initial steps of the dimerization process. For both reactions, it is assumed that the ethyl legs have been transformed into ethenyl groups through dehydrogenation reactions, the details of which are shown in the Supplementary Information. (**a**,**d**) Chemical stick models and (**b**,**e**) top and side view ball models for reactants (**IS**), transition states (**TS**), reaction intermediates (**Int**) and the monomer reaction final state (**FS**). (**c**,**f**) Energy profiles for the monomer cyclization and initial steps of the dimerization process, respectively. Free energy barriers are shown in parentheses and indicated by the dotted lines for **TS1** of each reaction, calculated by including vibrational enthalpy and entropy at 275 °C. Energies are given in units of eV.

From this starting point, the monomer electro-cyclization is followed by the actual ring-closing reaction, with an effective potential energy barrier of 1.82 eV as seen in Fig. 6a–c (the ring closing reaction is associated with several barriers, which are shown in Supplementary Fig. 8). Two dehydrogenation steps with relatively small energy barriers are required for the final aromatization process providing the isoindole unit. These results suggest the possibility to tune the reaction temperature such that the ethyl-to-ethenyl transformation is triggered, without activating the ring closuring reaction owing to its significantly higher energy barrier.

Next, we investigate the dimerization reaction, starting from two monomers that have experienced a complete dehydrogenation of the ethyl residues. The reaction is initiated by a [2+2] cycloaddition with an effective potential energy barrier of 1.04 eV (cf. Fig. 6d–f, **IS** to **Int2**). Notably, the [2+2] cycloaddition proceeds in a two-step mechanism, via an intermediate state (**Int1**) in which a carbon atom is chemically bonded to the surface. Importantly, the cycloaddition is just slightly exothermic with a reaction energy of −0.25 eV, providing a Boltzmann factor of ∼200 between **Int2** and **IS** at 275 °C, thereby substantiating the reversibility of the coupling reaction. This supports the suggested pathway in Fig. 5a, and further detailed in Supplementary Fig. 5, in which **IS** and **Int2** are in thermal equilibrium before the reaction proceeds in a Diels–Alder step. Future studies will unravel the information of the final steps of the dimerization.

In addition to potential energy barriers, we also considered the effect of zero-point and thermal contributions on the decisive steps of the monomer cyclization and dimerization by including vibrational enthalpy and entropy. Free energy barriers were evaluated at a temperature of 275 °C and are indicated for the initial monomer and dimerization steps in Fig. 4c,f, respectively. The monomer ring-closing reaction is slightly lowered by 0.16 eV, whereas the free energy barrier for the dimerization is 0.11 eV larger than the corresponding potential energy barrier. Supplementary Table 1 shows the individual contributions from vibrational enthalpy and entropy on the two barriers.

Although zero-point and thermal vibrational contributions have a small quantitative effect on barriers, both the potential energy and the free energy landscape unambiguously demonstrate that the decisive step for the dimerization has a significantly lower barrier than the monomer cyclization, with potential energy and free energy barrier differences of 0.72 and 0.51 eV, respectively. As a result, the monomer ring-closure will occur only if the dimerization is kinetically hindered, such as for extremely low coverage or in the absence of nearby non-reacted molecules. Hereby, the balance between diffusion and reaction barriers plays a crucial role, as the molecules need to meet to undergo the polymerization. These results explain why the monomer ring closure is observed for the deposition at the already heated substrate, in which situation the molecules experience the electrocyclic ring closure before meeting other species, while the intermolecular connections are formed when depositing the molecules at a room temperature substrate followed by thermal annealing allowing the precursors sufficient time to diffuse.

In summary, we have successfully introduced thermally tunable covalent reactions on surfaces to engineer quasi-unidimensional phthalocyanine tapes or self-assembled phthalocyanines by depositing molecular precursors on a well-defined Au(111) surface and regulating the substrate temperature. Our results open avenues to thermally control reaction pathways on surfaces, selecting intermolecular versus intramolecular reactions, and thus allowing to induce the growth of unprecedented polymeric heteroatomic nanoarchitectures or to produce monomeric reactions. DFT simulations corroborate the experimentally observed behaviour, demonstrating that the decisive step determining the reaction product has a lower free energy barrier for the dimerization compared with monomer intramolecular cyclization reaction. Our study discloses strategies to grow uni- and two-dimensional polymeric nano-architectures embedding heteroatomic monomers. Such systems bear prospects for molecular electronics, optoelectronics and photovoltaics.

## Methods

### Experiments

The experiments were performed in a custom-designed ultra-high vacuum system that hosts a low-temperature Omicron scanning tunnelling microscope, where the base pressure was below 5 × 10^−10^ mbar. All STM images were taken in constant-current mode with electrochemically etched tungsten tips, applying a bias (*V*_b_) to the sample and at a temperature of ∼80 K. The Au(111) substrate was prepared by standard cycles of Ar+ sputtering (800 eV) and subsequent annealing to 723 K for 10 min. OETAP molecules were synthesized according to the procedure described by Fitzgerad and co-workers^35^,^36^ and deposited by organic molecular-beam epitaxy from a quartz crucible held at 450 K onto a clean Au(111) at room temperature, if not stated otherwise. If necessary, in a subsequent step, the samples were annealed with a thermal gradient of 1 °C s^−1^ and kept at the desired temperature for 30 min. Next, they were cooled down to room temperature with a thermal gradient of −1 °C s^−1^ and finally transferred to the STM stage held at 77 K.

### Theory

Periodic DFT calculations were performed with the VASP code^37^, using the projector-augmented wave method^38^. Exchange-correlation effects were described by the version of the van der Waals density functional^39^ (vdWDF) introduced by Hamada^40^ denoted as rev-vdWDF2. Transition states were calculated using the climbing image nudged elastic band^41^ and the Dimer^42^ methods. STM simulations were carried out with the Tersoff–Hamann approximation^43^ using the implementation by Lorente and Persson^44^. Detailed information about DFT calculations are provided in Supplementary Methods.

## Additional information

**How to cite this article:** Cirera, B. *et al*. Thermal selectivity of intermolecular versus intramolecular reactions on surfaces. *Nat. Commun.* 7:11002 doi: 10.1038/ncomms11002 (2016).

## Supplementary Material

Supplementary Information Supplementary Figures 1-8, Supplementary Table 1, Supplementary Note 1, Supplementary Methods and Supplementary References

Supplementary Movie 1 Diffusion of on-surface synthesized polymeric phthalocyanines on Au(111)
