# Supplementary material for: Thermal selectivity of intermolecular versus intramolecular reactions on surfaces
Source: Nat Commun. 2016 Mar 11;7:11002. doi: 10.1038/ncomms11002 (PMC4793044; doi:10.1038/ncomms11002)
Supplement: Supplementary Information — Supplementary Figures 1-8, Supplementary Table 1, Supplementary Note 1, Supplementary Methods and Supplementary References [file ncomms11002-s1.pdf]

Supplementary Figures

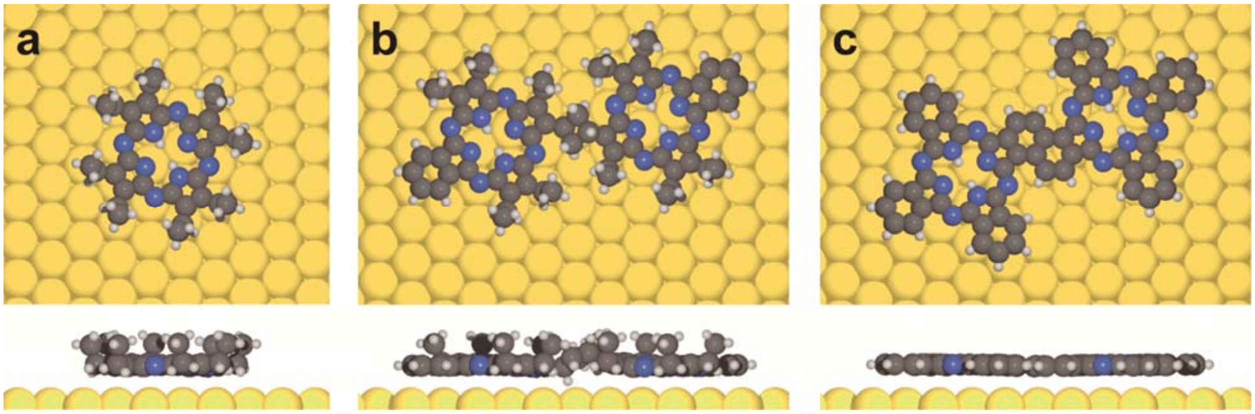

Supplementary Figure 1. Top and side views of optimized adsorption geometries of (a) OETAP monomer, (b) **1-L** reaction intermediate and (c) **3-L** reaction product.

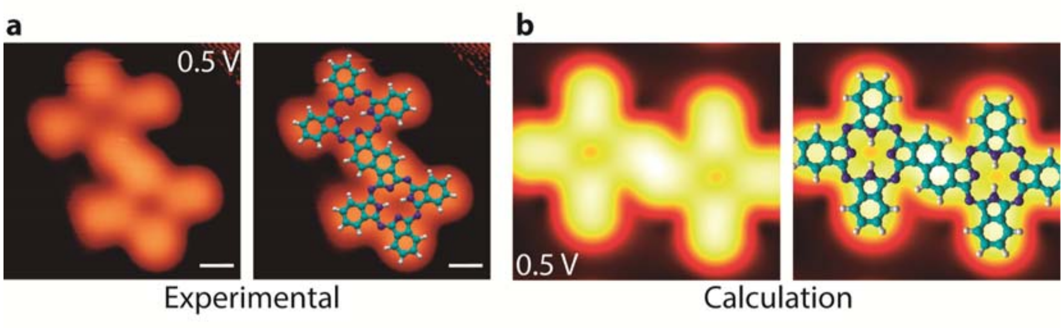

Supplementary Figure 2. STM and DFT simulated images of a **3-L** reaction product (L-type motif) at V_{bias} = 0.5V. Scale bars: 0.5 nm.

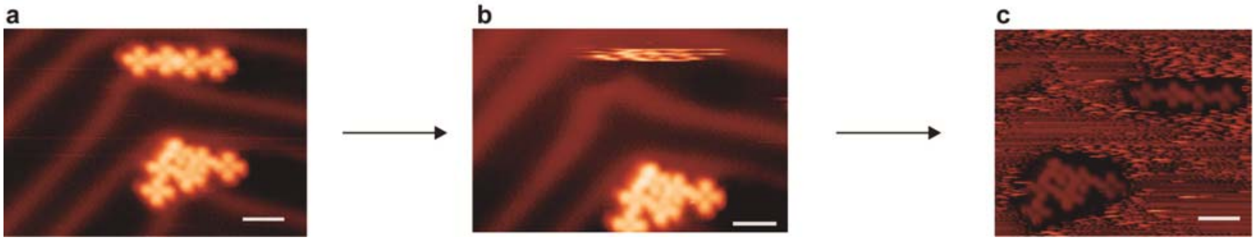

Supplementary Figure 3. Displacement of a phthalocyanine oligomer under perturbative scanning conditions. Tunneling parameters: **a-b**, V_b = -1.5 V, I_t = 15 pA; **c**, V_b = -0.5 V, I_t = 150 pA. Scale bars: 1 nm.

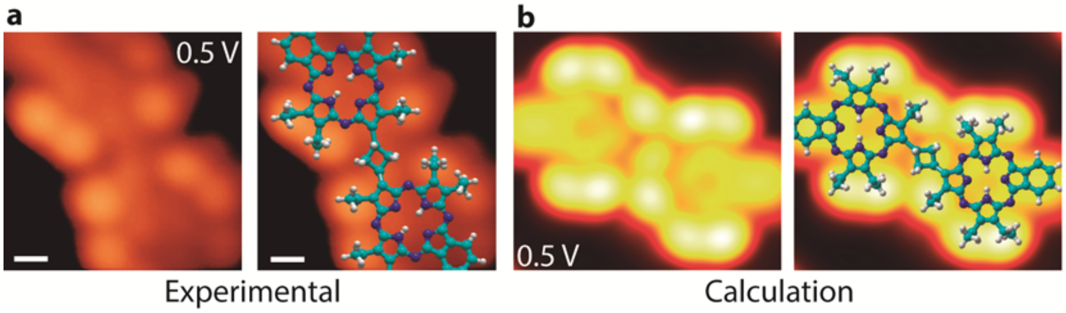

Supplementary Figure 4. STM and DFT simulated images of **1-L** reaction intermediate at V_{bias} = 0.5V. Scale bars: 0.3 nm.

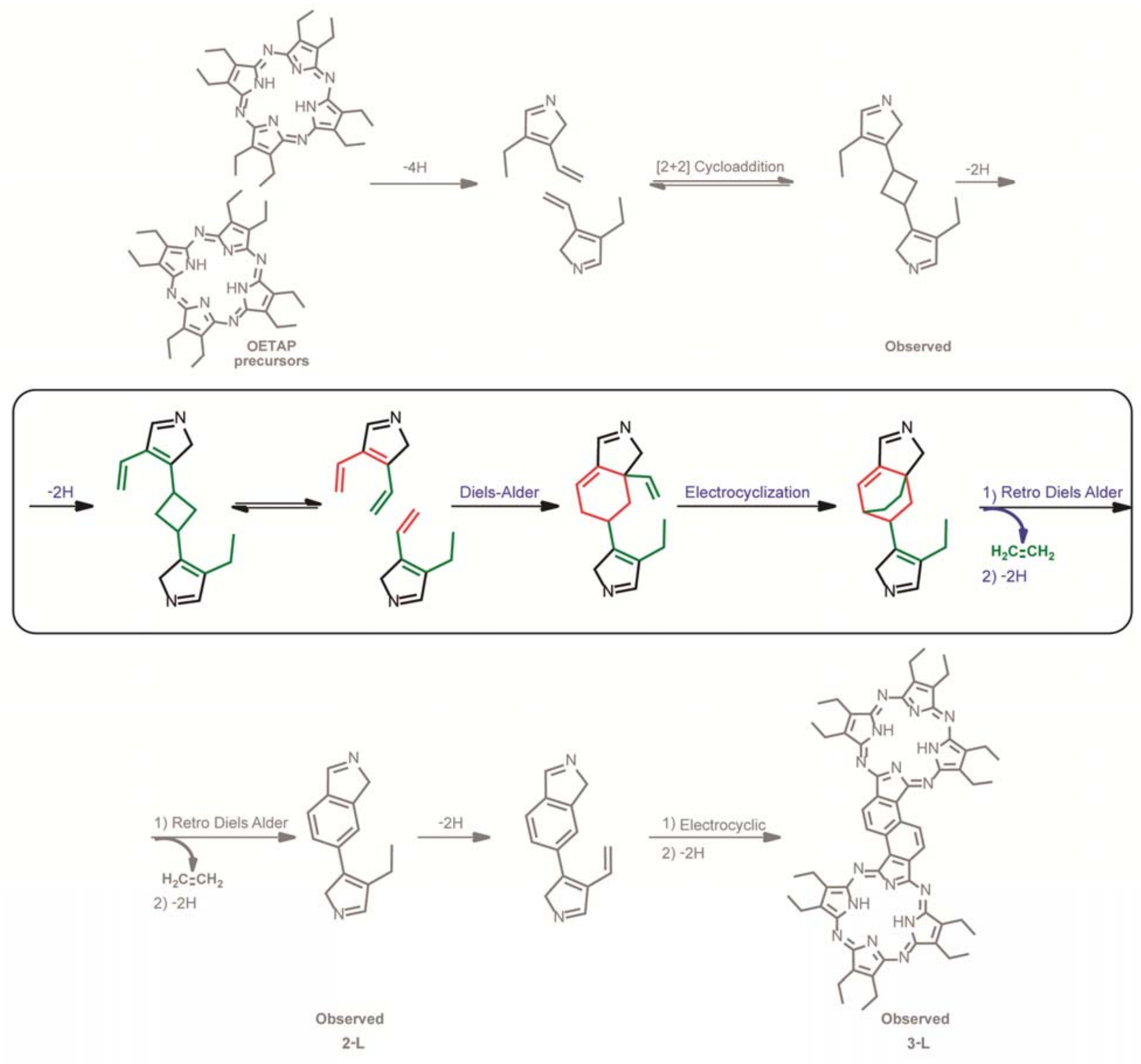

Supplementary Figure 5. Suggested reaction mechanisms for the surface-assisted synthesis of L-type phthalocyanine dimer on Au(111). The L-type linking motif is a result of an interaction between ethyl moieties on the opposite side of opposite OETAP precursors in the first step of the dimerization reaction. The faded steps represent those already depicted in the main text (Figure 5a); the transformation of intermediate **1-L** to intermediate **2-L**, both observed by STM imaging, is highlighted.

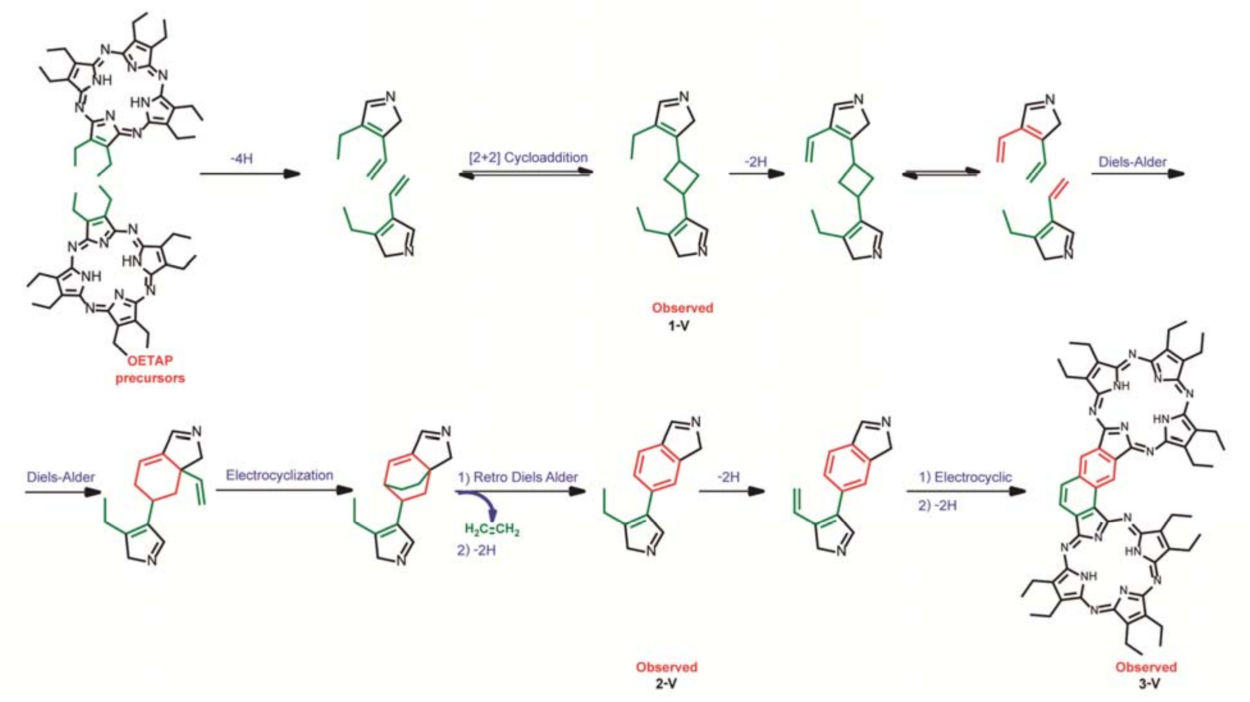

Supplementary Figure 6: Suggested reaction mechanisms for the surface-assisted synthesis of V-type phthalocyanine dimer on Au(111). The V-type linking motif is a result of an interaction between ethyl moieties on the same side of opposite OETAP precursors in the first step of the dimerization reaction.

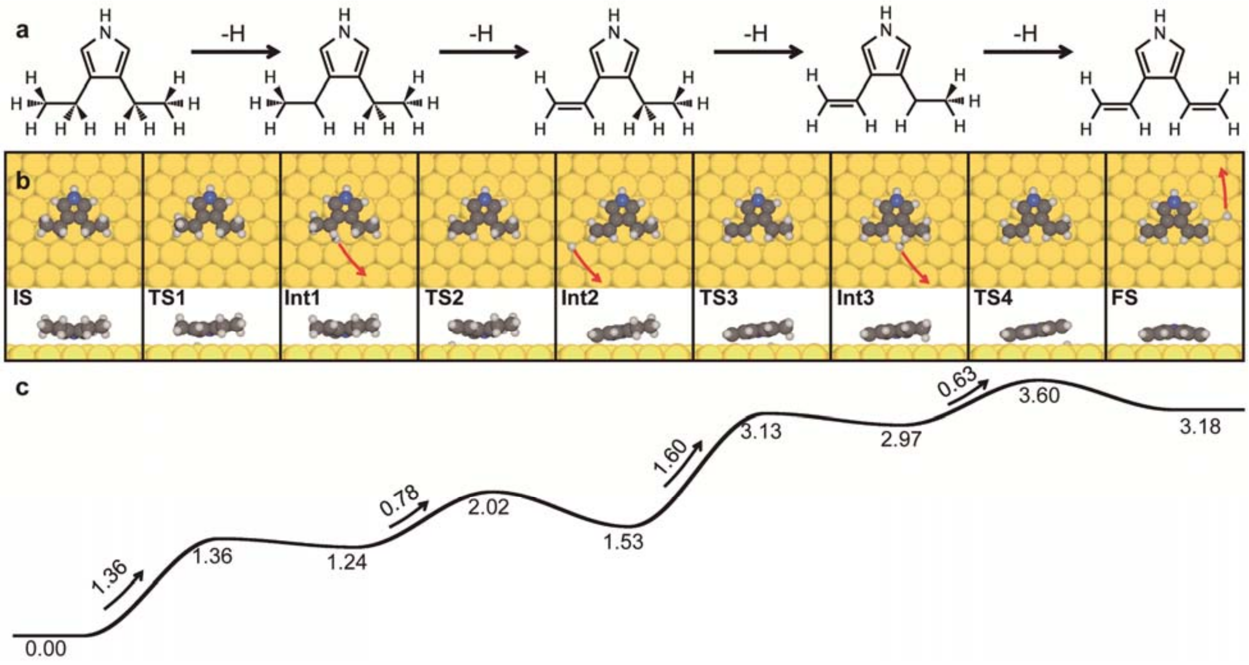

Supplementary Figure 7. The ethyl-to-ethenyl transformation of the model molecule on Au(111), which initiate both the monomer cyclization and dimerization reaction shown in the main manuscript. (a) Chemical models of initial state (IS), intermediates (Int) and final state (FS); (b) top and side views of the on-surface structures (including transition states, TS); and (c) the corresponding energy profile. Energies are given in units of eV.

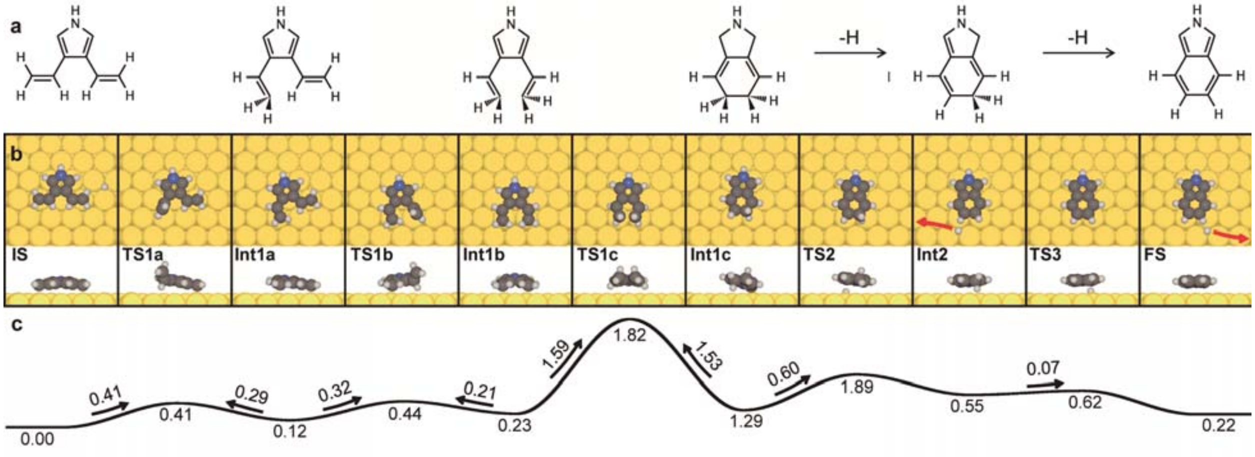

Supplementary Figure 8. Complement to Figure 6 in the manuscript: The full monomer cyclization reaction, starting from the ethyl-to-ethenyl model molecule (Supplementary Figure 7), explicitly showing the rotations of the two ethenyl groups (**IS**-to-**Int1a** and **Int1a**-to-**Int1b**), which are part of the overall ring closure (**IS**-to-**Int1c**).

Supplementary Tables

Supplementary Table 1. Contributions from potential (electronic) energy (E_{elec}), vibrational enthalpy (H_{vib}) and vibrational entropy (S_{vib}) to the free energy (G), calculated at a temperature of 275 °C for **IS** and **TS1** for the monomer cyclization and dimerization, shown in Figure 6. For each term, the difference between **TS1** and **IS** is indicated.

	E_{elec} (eV)	H_{vib} (eV)	$- TS_{vib}$ (eV)	$G = E_{elec} + H_{vib} - TS_{vib}$ (eV)
Monomer cyclization				
**IS**	0	4.76	-1.70	3.06
**TS1**	1.82	4.69	-1.78	4.72
Δ(**TS1-IS**)	1.82	-0.07	-0.08	1.66
Dimerization				
**IS**	0	9.53	-3.49	6.04
**TS1**	1.04	9.49	-3.34	7.19
Δ(**TS1-IS**)	1.04	-0.04	+0.15	1.15

Supplementary Notes

Supplementary Note 1: Tentative reaction mechanisms

We propose that following dehydrogenation of one remaining ethyl group in **1-L/V**, an aromatic equilibrium is established due to the constrained four-membered ring. Subsequently, a [4+2] cycloaddition or Diels-Alder reaction occurs, where the motif containing the alkene arm acts as a diene and the dienophile role is performed by the dehydrogenated ethyl group on the opposite molecule [1] (in red in Supplementary Figures 5 and 6). Then a pericyclic rearrangement leads to the next intermediate, which forms intermediate **2-L/V** after undergoing a retro Diels-Alder reaction with loss of ethylene. The proposed mechanism agrees with a loss of two carbon atoms, necessary to explain the final merging motif between the two original OETAP precursors.

Supplementary Methods

Details of DFT calculations

Periodic density functional theory (DFT) calculations were performed with the VASP code [2], using the projector-augmented wave method [3], and a kinetic energy cutoff of 400 eV. Exchange-correlation effects were described by the version of the van der Waals density functional (vdWDF) [4] introduced by Hamada [5] denoted as rev-vdWDF2, which has shown to accurately describe adsorption of different polycyclic aromatic hydrocarbons on Au(111) [6]. The Au(111) surface was approximated by a four layered slab separated by a vacuum region of 15 Å. The surface reconstruction was not taken into account, as this has shown to have very little effect on the surface reactivity [7]. All atoms, except for the bottom two layers of the Au slab, were structural optimized of local minima were performed until the forces acting on the atoms were smaller than 0.01 eV/Å. For the calculations of the monomer cyclization and the dimerization, $p(6\times6)$ and $p(6\times8)$ surface unit cells were used, respectively, together with a 4×4 k-point samplings.

Transition states were calculated using a combination of the climbing image nudged elastic band (CI-NEB) [8] and Dimer methods [9]. For the CI-NEB calculations the number of images was adjusted specifically for each transition-state calculation such that the tangent along the path was well described, using typically 15−20 images. The CI-NEB method was used to find a rough estimate of the transition state, which was used as input for the Dimer method. The structural optimizations of transition states was performed until the forces acting on the atoms on the central images, in the Dimer method, were smaller than 0.02 eV/Å.

Vibrational enthalpy and entropy was calculated from the vibrational frequencies at local minima and transition states, within the framework of the harmonic approximation and obtained using finite differences.

STM simulations were carried out with the Tersoff-Hamann approximation [10] using the implementation by Lorente and Persson [11].

Supplementary References

[1] Handbook of Porphyrin Science: With Applications to Chemistry, Physics, Materials Science, Engineering, Biology and Medicine, Volume 17: Synthetic Developments (Part II), chapter 76, pp. 87-97. Edited by: Karl M. Kadish, Kevin M. Smith, Roger Guilard. World Scientific, 2012.

[2] Kresse, G. & Furthmüller, J. Efficient iterative schemes for ab initio total-energy calculations using a plane-wave basis set. *J. Phys. Rev. B* **54**, 11169−11186 (1996).

[3] Blöchl, P. E. Projector augmented wave method. *Phys. Rev. B* 50, 17953−17979 (1994).

[4] Dion, M. *et al*. Van der Waals Density Functional for General Geometries. *Phys. Rev. Lett.* **92**, 246401−246404 (2004).

[5] Hamada, I. van der Waals density functional made accurate. *Phys. Rev. B* **89**, 121103(R)–121107(R) (2014).

[6] Björk, J. & Stafström, S. Adsorption of large hydrocarbons on coinage metals: A van der Waals density functional study. *ChemPhysChem* **15**, 2851–2858 (2014).

[7] Hanke, F. & Björk, J. Structure and local reactivity of the Au(111) surface reconstruction. *Phys. Rev. B.* **87**, 235422–235427 (2013).

[8] Henkelman, G., Uberuaga, B. P. & Jónsson, H. A climbing image nudged elastic band method for finding saddle points and minimum energy paths. *J. Chem. Phys.* 113, 9901−9904 (2000).

[9] Kästner, J. & Sherwood, P. Superlinearly converging dimer method for transition state search. *J. Chem. Phys.* **128**, 014106−014111 (2008).

[10] Tersoff, J. & Hamann, D. R. Theory and Application for the Scanning Tunneling Microscope. *Phys. Rev. Lett.* **50**, 1998-2001 (1983).

[11] Lorente, N. & Persson, M. Theoretical aspects of tunneling-current-induced bond excitation and breaking at surfaces. *Farad. Discuss.* **117**, 277-290 (2000).
